# Effects of running on adiponectin, insulin and cytokines in cerebrospinal fluid in healthy young individuals

**DOI:** 10.1038/s41598-018-38201-2

**Published:** 2019-02-13

**Authors:** M. Schön, Z. Kovaničová, Z. Košutzká, M. Nemec, M. Tomková, L. Jacková, D. Máderová, L. Slobodová, P. Valkovič, J. Ukropec, B. Ukropcová

**Affiliations:** 1 Institute of Experimental Endocrinology, Biomedical Research Center, Slovak Academy of Sciences, Dúbravská cesta 9, 94505 Bratislava, Slovakia; 2 Institute of Pathological Physiology, Faculty of Medicine, Comenius University, 81108 Bratislava, Slovakia; 3 2nd Department of Neurology, Faculty of Medicine, Comenius University, University Hospital Bratislava, 83305 Bratislava, Slovakia; 4 Faculty of Physical Education and Sports, Comenius University, 81469 Bratislava, Slovakia

## Abstract

Exercise can prevent the sedentary lifestyle-related risk of metabolic and cognitive decline, but mechanisms and mediators of exercise effects on human brain are relatively unexplored. We measured acute exercise-induced changes in adiponectin, insulin and other bioactive molecules in cerebrospinal fluid (CSF) and serum from young lean individuals. Samples of serum and CSF were obtained before and 1-h after the 90-min run (75–80% HRmax; maximal heart rate), additional serum was taken at finish-line. Body composition, physical fitness, metabolic rate, cognitive functions, food preference, glucose, insulin and albumin were measured. The spectrum of 174 cytokines was assessed by protein arrays, adiponectin was also determined by ELISA and immunoblotting. CSF adiponectin decreased post-exercise by 21.3% (arrays) and 25.8% (ELISA) (p < 0.009). Immunoblotting revealed reduction in a low-molecular-weight-adiponectin (p < 0.005). CSF adiponectin positively correlated with CSF/serum albumin ratio (p < 0.022), an indicator of blood-brain-barrier permeability. CSF and serum adiponectin were positively associated with memory and running-induced changes in insulinemia and CSF insulin. Additionally, running modulated CSF levels of 16 other cytokines. Acute running reduced CSF adiponectin and modulated insulin and albumin in CSF and serum. Associations of adiponectin with memory and metabolism indicate the potential role of this bioactive molecule in mediating exercise-induced adaptive response in human brain.

## Introduction

Regular exercise represents an effective prevention and treatment of metabolic and neurodegenerative diseases^1^. Mechanisms mediating exercise-induced health benefits in both periphery and the brain include changes in body composition^2^ and energy metabolism^3^, reduction of systemic inflammation^4^ and secretion of bioactive molecules^5,6^. Physical activity can influence energy balance by increasing energy expenditure and by modulating appetite/energy intake^7^. Signals controlling balance between appetite and energy metabolism arise from both fat and lean body mass, and are in principle energy sensing mechanisms regulated by energy intake and physical exertion^8^.

Benefits of exercise are, at least to an extent, mediated by exerkines, bioactive molecules released into circulation during and/or after exercise^9^. Contracting skeletal muscles have been identified as a source of myokines synchronizing processes of systemic adaptation to exercise^5,10^. Evidence from animal studies indicates that other tissues also produce molecular mediators of exercise-induced benefits. Yau *et al*. showed that adipose tissue-derived adiponectin could pass the blood-brain barrier and decrease depression-like behaviour while adiponectin deficiency diminished both exercise-induced increase in hippocampal neurogenesis and antidepressogenic effects of exercise in mice^11^. Neumeier *et al*. used the relationship between CSF/serum ratios of albumin and adiponectin to indicate changes of blood-brain-barrier or blood-CSF-barrier permeability, which could be implicated in adiponectin transport^12^. Adiponectin receptors are located on the hypothalamic neurons, which are involved in metabolic regulation, such as POMC and NPY/AgRP neurons^13,14^. Moreover, leptin and adiponectin synergistically modulate neuronal excitability of melanocortin neurons to regulate energy balance and glucose metabolism^15^. Liver overexpression of adiponectin protected mice from high calorie diet-increased weight gain and insulin resistance by modulating whole body energy expenditure, preventing thus the premature death^16^. Adiponectin, well-known for its insulin-sensitizing, antiinflammatory and antidiabetogenic properties, is in this respect one of the best described adipokines^17^. Neuroprotective actions of adiponectin were observed in murine brain^18–21^, while adiponectin deficiency in mice was associated with reduced dendritic lenght/branching/spine density of neurons and decreased neurogenesis, which could be reversed by intracerebroventricular infusion of adiponectin^21^. Aged adiponectin-deficient mice display spatial memory and learning impairments, fear-conditioned memory deficits as well as anxiety. These mice even develop Alzheimer’s disease (AD) pathology, which is associated with hippocampal insulin resistance^20^. On the other hand, cognitive dysfunction and Alzheimer’s disease in humans are linked to higher serum adiponectin levels^22–25^, although evidence regarding CSF adiponectin levels in Mild Cognitive Impairment (MCI)/AD patients remains controversial^23,24^. Taken together, evidence mostly from animal studies suggest that adiponectin is involved in the regulation of brain plasticity and energy metabolism.

Cerebrospinal fluid plays a key role in brain homeostasis, providing protection, clearing waste and maintaining levels of ions/metabolites/nutrients/metals^26^. A range of bioactive molecules can be detected in CSF, including cytokines, which are being explored as putative biomarkers of neurologic diseases^27,28^. Kierkegaard *et al*. showed that 12-weeks of high-intensity training did not change proinflammatory cytokines in CSF of patients with multiple sclerosis, despite an improvement in the clinical state^29^. In one more study, an acute exercise bout induced an increase in Vascular Endothelial Growth Factor (VEGF) levels^30^ as well as modulated the spectrum of metabolites^31^ in CSF of patients with chronic hydrocephalus. Physical activity was shown to modulate CSF biomarkers of Alzheimer’s disease in seniors with mild cognitive impairment^32^. It can be assumed that changes in bioactive molecules in CSF can be associated with both benefits of exercise and with the pathogenesis of neurodegeneration. Moreover, the specific molecular fingerprint in CSF could be used as an early marker for the increased risk of neuropathology.

The aim of our study was to investigate effects of running on levels of CSF and/or serum cytokines, insulin and albumin in young healthy individuals in association with cognitive functions, metabolism, food preference and physical fitness, in order to identify putative molecular mediators of exercise-induced effects on the brain.

## Results

### Characteristics of the study population

Detailed characteristics of the study population, young healthy individuals (amateur runners), are presented in Table 1.

**Table 1:** Characteristics of the study population.

	Volunteers who completed both baseline and exercise days	Volunteers who completed the exercise day only
Age (years)	22.7 ± 2.9	24.6 ± 5.1
Gender (M/F)	6/3	6/5
BMI (kg/m^2^)	23.1 ± 2.0	22.7 ± 1.8
Body weight (kg)	71.9 ± 7.0	69.1 ± 8.0
Body fat (%)	21.3 ± 6.5	21.3 ± 6.7
Muscle mass (%)	37.8 ± 5.4	37.6 ± 6.0
Visceral fat (%)	4.8 ± 1.9	4.4 ± 1.5
*Waist circumference (cm)	78.7 ± 7.2	75.7 ± 6.2
*Respiratory quotient (VCO_2_/VO_2_)	0.83 ± 0.05	0.82 ± 0.05
*Resting energy expenditure (kcal/24 h)	1497.0 ± 222.9	1520.8 ± 333.8
*${\dot{{\rm{V}}}O}_{2}$ max (mlO_2_/kg_BW_/min)	42.2 ± 6.0	41.1 ± 6.7
*HRmax (1/min)	168.7 ± 19.5	170.4 ± 20.1

BMI, Body Mass Index; ${\dot{{\rm{V}}}O}_{2}$ max, maximal aerobic capacity; HRmax, maximal heart rate. Resting energy expenditure and respiratory quotient were assessed by indirect calorimetry, ${\dot{{\rm{V}}}O}_{2}$ max by cycle spiroergometry, data are expressed as mean ± SEM. *Data available in 6(3/3) and 8(3/5) individuals.

### An acute bout of intense aerobic exercise modulated cytokine levels in CSF

**Figure 1:**
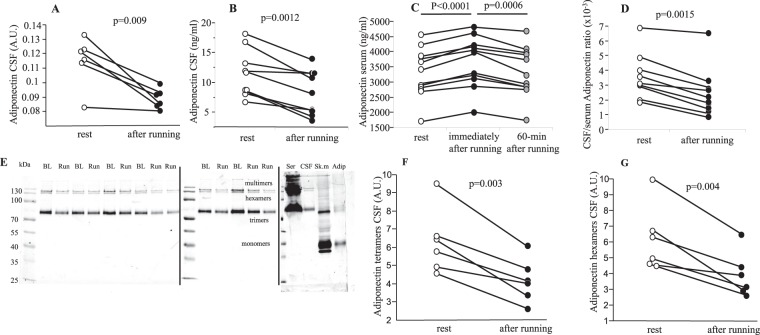
An acute bout of aerobic exercise (90-min run) modified the levels of adiponectin in cerebrospinal fluid and serum of healthy trained volunteers. Adiponectin levels assessed by (**A**) protein arrays (CSF, n = 6), normalized to reference proteins; (**B**) ELISA (CSF, n = 9), (**C**) ELISA (serum, n = 11), (**D**) ELISA (CSF/serum ratio, n = 9), (**E**,**F**,**G**) immunoblotting (CSF, n = 6/8, B/R), in all CSF samples (CSF, cerebrospinal fluid; BL, baseline CSF sample; Run, CSF taken 30–60-min after an 90-min run; Ser, serum (load 4 ml); Sk.m, skeletal muscle (load 40 mg); Adip, human subcutaneous adipose tissue (load 20 mg), Black vertical lines delineate the boundaries between the three separate full-length blots performed under the identical experimental conditions. Statistical differences were analysed using paired Student’s t-test, A.U. normalized signal intensity.

The effect of an acute bout of intense aerobic exercise (90-min run, ~75% HRmax) on the levels of 174 cytokines was explored in 6 paired CSF samples from young healthy volunteers (M/F, 3/3), using protein arrays. Running induced a 21.3% decrease of adiponectin (Fig. 1A) and ≥10% decrease of IL-18Rβ and PDGF-AA levels in CSF. There was also >5% decrease in IL5Rα, LAP, MIG, MMP-13, TGFβ2, Tie-1, Activin-A, IL-18 binding protein α and IGF-II levels in CSF, and a small (<5%) but significant running-induced increase in IL-2 and a decrease in IL13Rα, TGF-b3, MPIF-1 and thrombopoietin (p < 0.05 for all). All acute exercise-induced changes in CSF levels of 174 cytokines are listed in the Supplementary Table 1.

### Acute aerobic exercise modified adiponectin and insulin levels in CSF and circulation

The running-induced reduction in CSF adiponectin assessed by protein arrays was confirmed by ELISA (−25.8%, p = 0.0012) (Fig. 1A,B). Importantly, immunoblotting revealed 33.3% decrease of adiponectin trimers and 38.2% decrease of adiponectin hexamers in CSF (Fig. 1E–G). The adiponectin trimers/hexamers ratio in CSF was not affected by an acute bout of running. Running also caused 10.5% immediate increase of serum adiponectin (Fig. 1C), which was followed by a complete normalisation after 1-h recovery (Fig. 1C). There was a 35.6% decrease in CSF/serum adiponectin ratio after running (Fig. 1D), suggesting an acute running-induced reduction of the BBB (blood-brain barrier) permeability for adiponectin, uptake of adiponectin by the brain or running-enhanced secretion of CSF. The levels of adiponectin in CSF represented only 0.35% and 0.23% of serum adiponectin at the baseline and post-exercise states, respectively.

Insulin levels in CSF were reduced by average 22.4% (the range 6.9–38.9%) in all but one individual (n = 5, p = 0.032; n = 6, p = 0.299). Acute exercise reduced also levels of serum insulin 1-h post-recovery (n = 6, 6.78 ± 3.12 vs. 4.48 ± 2.65 mIU/l, p = 0.019), but not immediately after the run (p = 0.376), indicating improvements in insulin sensitivity (lowering HOMA-IR; homeostatic model assessment - insulin resistance) 1-h post-exercise (1.56 ± 0.86 vs. 0.92 ± 0.59, p = 0.018), but not immediately after the run (p = 0.171).

### Effect of an acute aerobic exercise on the blood-brain barrier permeability markers

**Figure 2:**
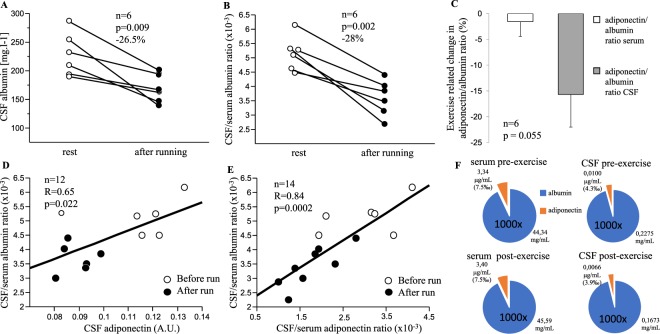
The effect of an acute exercise bout (90-min run) on (**A**) albumin in cerebrospinal fluid (CSF); (**B**) CSF/serum albumin ratio and (**C**) adiponectin/albumin ratio in serum and CSF. CSF/serum albumin ratio, the marker of blood-brain barrier permeability, is associated with adiponectin levels in CSF (**D**) and CSF/serum adiponectin ratio (**E**). Unlike in serum, adiponectin/albumin ratio was lower in CSF post-exercise (**F**). Statistical differences were analysed using paired student’s T-test and Paerson linear correlation analysis, A.U. normalized signal intensity.

Running induced 26.5% decrease in CSF albumin content and 28% decrease in CSF/serum albumin ratio (Fig. 2A,B), indicating an exercise-induced decrease in blood-brain barrier permeability. Running induced an immediate increase of serum albumin (7.2%, 43.8 ± 3.7 vs. 47.0 ± 2.7, p = 0.043), which was followed by a return to the baseline levels 1-h post-exercise (44.4 ± 3.0, p = 0.027). CSF adiponectin, which displayed by far the greatest response to an acute running bout when compared to other cytokines (Supplementary Table 1), was positively associated with CSF/serum albumin ratio (Fig. 2D). Moreover, there was a positive correlation between CSF/serum albumin ratio and CSF/serum adiponectin ratio (Fig. 2E). However, we did not observe any associations between CSF/serum albumin ratio and the levels of other cytokines modified by a 90-min run in CSF.

The level of albumin in CSF represented 0.51% of albumin in serum at baseline and 0.34% after an acute bout of exercise. The ratio of albumin in CSF and serum therefore decreased by 27.6%. Whereas, the level of adiponectin in CSF represented 0.35% of adiponectin in serum at baseline and 0.23% after run and the CSF-to-serum adiponectin ratio decreased by 34.3% after run compared to baseline (p = 0.005). The acute exercise-induced decrease of adiponectin in CSF was 6.16% greater than that of albumin. Moreover, CSF adiponectin/albumin ratio dropped post exercise by 15.6% while that of serum remained unchanged by an acute intense running bout (Fig. 2C), providing another indication that some of the adiponectin is being extracted by the brain tissues (Fig. 2C,D).

### Associations of CSF and serum adiponectin with cognitive functions, physical fitness, metabolism and nutritional preference

**Figure 3:**
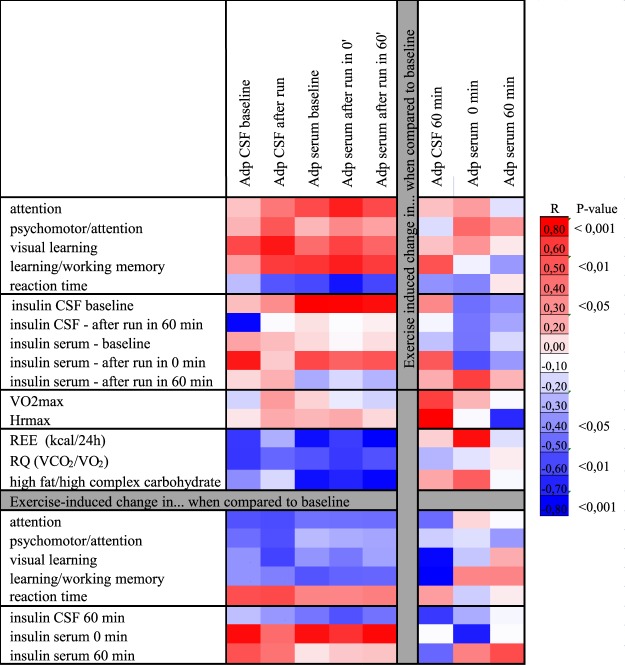
Heat map. Associations between adiponectin levels in CSF and serum assessed by ELISA and cognitive functions, metabolic parameters, physical fitness and nutrient preference.

The post-run CSF adiponectin levels were positively associated with both visual learning and learning/working memory score. These two cognitive parameters were also positively associated with serum adiponectin measured at baseline (pre-run), immediately after and 1-h after the run, as well as with the levels of CSF insulin (R = 0.566, p = 0.034). In contrast, reaction time at the identification task was negatively associated with serum adiponectin levels measured immediately post-run (Fig. 3). Stepwise regression model evaluating effects of serum levels of adiponectin, glucose, insulin and albumin revealed that serum adiponectin was the best predictor of the learning/working memory, explaining more than 33% of its variability. An acute bout of running did not induce a significant change in cognitive tests scores in healthy young individuals (p > 0.1 for all).

The running-induced decrease in CSF adiponectin was less pronounced in individuals with high fitness levels as determined by heart rate at the peak of oxygen consumption during cycle spiroergometry (HRmax) (Fig. 3).

Basal (pre-run) adiponectin levels in both CSF and serum were negatively associated with resting metabolic rate. Serum adiponectin levels were positively associated with baseline CSF insulin, irrespective of the acute intense exercise and exercise-induced change in insulinemia was positively associated wih baseline CSF and serum adiponectin levels and negatively with the immediate running-induced change of serum adiponectin (Fig. 3). CSF insulin level was positively associated with the CSF content of adiponectin trimers (R = 0.604, p = 0.023) and hexamers (R = 0.620, p = 0.018). In contrast, there was a trend towards negative associations between basal levels of IL-18Rβ and PDGF in CSF and the running-induced change in insulinemia (n = 6, R = −0.75, p = 0.084; R = −0.79, p = 0.059) and HOMA-IR (n = 6, R = −0.75, p = 0.088; R = −0.78, p = 0.069), suggesting specific regulation and possibly distinct roles of different exercise-regulated cytokines in the central nervous system.

Serum adiponectin was negatively associated with the preference for food rich in fat and complex carbohydrates (food preference questionnaire). The associations of CSF & serum levels of adiponectin (absolute values and running-induced change) with CSF & serum levels of insulin, physical fitness, resting metabolism and food preference are presented in Fig. 3.

## Discussion

Exercise-induced inter-organ crosstalk includes communication between periphery and the brain^10^. Evidence from studies in humans is, however, sparse^29,30,32–34^. Acute bout of intense aerobic exercise specifically modulates levels of 17 out of 174 cytokines we have measured in cerebrospinal fluid (CSF) of healthy young volunteers. Furthermore, we identified adiponectin as the cytokine displaying the greatest exercise-induced change in CSF. Using three different methodologies (protein arrays/ELISA/immunoblotting), we consistently observed 21.3–38.2% reduction of CSF adiponectin levels after 90-min run in healthy young physically active volunteers. In agreement with previous studies in humans, and in contrast to serum, majority of adiponectin we detected in CSF was of low molecular weight (LMW)^35^. CSF adiponectin positively correlated with CSF/serum albumin ratio and represented less than 0.35% of the serum adiponectin, which is in agreement with findings from others^12,35^. The ratio between trimeric and hexameric forms of adiponectin in CSF was not affected by an acute bout of running.

In addition, we observed >10% decrease in CSF levels of IL-18Rβ and PDGF, involved in the regulation of feeding in mice^36^ and in neuronal plasticity and embryonic development of the nervous system, respectively^37,38^. Other exercise-modulated cytokines in CSF are known to be involved in neuroprotection (IGF-II)^39^ and lipid metabolism (TGFβ)^40^. However, CSF levels of neurotrophins BDNF, VEGF and IGF-1, involved in exercise-induced neurogenesis/brain plasticity^41,42^, did not change after a single bout of long-distance running. There is a lack of evidence regarding effects of exercise on CSF levels of these neurotrophins in humans. An increase of VEGF in CSF, sampled 1-3-h after 20-min of moderate-intensity aerobic-strength upper-body exercise in elderly patients with hydrocephalus has recently been reported^30^. Differences in the study population, type/duration/intensity of exercise and timing of CSF sampling might contribute to different results.

The modest 10.5% increase of serum adiponectin was found immediately after the 90-min run, and it returned to baseline 1-h post-exercise. Effects of acute exercise on serum/plasma adiponectin remain controversial. This might be attributed to different exercise protocols as well as distinct study populations, but changes were always moderate or missing^43–47^. The possibility that acute exercise-induced change of serum adiponectin was due to haemoconcentration cannot be ruled out.

Exercise-induced changes in CSF levels of adiponectin and/or other cytokines could be due to changes in BBB/BCB (brain cerebrospinal fluid barrier) permeability, increased CSF turnover^48^ and/or increased adiponectin/cytokine uptake into the brain. Thus, we assessed the effect of a 90-min run on BBB/BCB permeability, using CSF/serum albumin ratio as a biomarker^49–52^. Post-run CSF/serum albumin ratio was markedly reduced, indicating lower BBB permeability or increased CSF flow/secretion/turnover^48^. This assumption was corroborated by the observation that changes in CSF/serum adiponectin ratio were paralleled by changes in CSF/serum albumin ratio, as described also by Neumeier *et al*.^12^. Others observed increased BBB permeability in response to acute aerobic exercise (short-term forced swimming) in specific brain regions in rats, using Evans blue^53^ and in humans, using serum S100^54,55^. On the other hand, Roszkowski *et al*. did not report increased BBB permeability in mice in response to acute swimming^56^.

LMW-adiponectin can cross the BBB barrier^13^ and its level in CSF is 1000-times lower than in the blood^35^. CSF levels of adiponectin represented <0.5% of serum adiponectin levels. Neumeier *et al*. concluded that adiponectin in CSF was far below levels expected by the baseline BBB permeability assessed by CSF/serum albumin ratio, possibly indicating an uptake of adiponectin by specific brain regions^12^, such as hippocampus or hypothalamus, which are known to express adiponectin receptors^11,15^. In mice, the intracerebroventricular injection of adiponectin did not result in any apparent leakage of adiponectin from cerebrospinal fluid to plasma, also supporting an active uptake of adiponectin by the brain^57^.

Adiponectin is an insulin-sensitizing, antiinflammatory adipokine^58^, stimulating muscle mitochondrial biogenesis and fatty acid oxidation^59,60^. Systemic and intracerebroventricular administration of adiponectin was shown to regulate food intake^13,61^, energy^57^ and glucose metabolism^62^ in mice. We found that CSF and/or serum adiponectin positively correlated with a running-induced change in serum insulin and HOMA-IR, suporting the role of adiponectin in regulating acute metabolic response to exercise. Regular exercise enhances insulin sensitivity in hypothalamus^63^. However, evidence on acute effects of exercise on brain/CSF insulin levels in humans is limited. CSF insulin levels were significantly decreased in 5 out of 6 individuals, indirectly indicating exercise-induced insulin uptake/action, possibly in those brain regions^64^, which might induce neuroprotection^65^, modulate feeding behaviour and cognitive functions^64^. Bioactive molecules in the peripheral circulation or CSF, including nutrients and adipose tissue-derived molecules, can modulate activity of hypothalamic areas involved in appetite regulation^66^. We observed a strong negative association between serum but not CSF adiponectin and preference for food rich in fat and complex carbohydrates. The running-induced change in adiponectin levels in CSF was negatively associated with a preference for food containing histidin, a precursor for dipeptide carnosine, known to have antioxidant, anti-glycation, chelating and pH buffering properties, and docosahexaenoic acid, a major fatty acid in the brain, involved in the brain development, neuronal functions, cognition and synaptic plasticity (data not shown). These findings support the role of peripheral signals in modulating appetite and energy metabolism in humans, as exemplified in animal models^15,67^.

Exercise-induced benefits for the brain, including increased neurogenesis, brain plasticity, improved cognitive functions and antidepressogenic effects have been described in mice^68–70^ and in humans^71–74^. Yau and colleagues showed that adiponectin could mediate some of the antidepressogenic effects of exercise via stimulating hippocampal neurogenesis^11^. Intracerebroventricular injection of adiponectin reduced proinflammatory cytokine production by microglia and blocked depressive-like behavior in corticosterone-treated mice^75^. Neuroprotective actions of adiponectin in the brain have been observed in several animal studies. Chronic adiponectin deficiency was associated with cognitive decline and AD-like pathogenesis in aged mice^20^. Administration of adiponectin prevented kainic acid-induced BBB breakdown and hippocampal cell death^19^ and increased synaptogenesis/dendritic complexity/neurogenesis in hippocampus (dentate gyrus) of adult mice^21^ and adiponectin receptor agonist was shown to influence synaptic plasticity in hippocampus^76^. Administration of osmotin, the plant homologue of mammalian adiponectin, attenuated β42-amyloid-induced memory impairment in the murine hippocampus^77^. On the other hand, ageing and neurodegeneration were linked to the altered adiponectin levels in CSF^23,24^ and circulation^22,25^. Lower plasma adiponectin was associated with a greater risk of cognitive impairment in men^78^. Adiponectin receptors were found in brain areas involved in cognition and a role of adiponectin in neuronal plasticity have been envisaged in patients with neurodegenerative diseases/cognitive dysfunction^23,24^.

Using young cognitively healthy physically active individuals for this pilot study precluded us from observing any immediate improvements in cognitive functions after high-intensity aerobic exercise. This might be due to the young age of our study population and/or exhaustion at the time of post-exercise testing. The evidence on the acute effects of exercise on cognition is rather controversial as it highly depends on the type of exercise, study population and cognitive domains that are evaluated^41^. Others have also failed to observe effects of acute high-intensity exercise on reaction time and attention (metaanalysis)^79^. Our intention, however, was to investigate putative associations between exercise-modulated adiponectin/cytokines and cognition. Importantly, we have observed that levels of CSF and/or serum adiponectin correlated positively with visual learning and learning/working memory, attention and reaction speed in young, healthy, physically active individuals. Serum adiponectin was in fact the best predictor of learning/working memory, explaining 33% of its variability, independent on serum levels of insulin, albumin and glucose. Taken together, this evidence supports the role of adiponectin in modulating cognitive performance, which is in agreement with studies exploring the link between adiponectin and cognition in animal models and humans with neurodegenerative diseases^20,21^ as well as its role in mediating effects of exercise on the brain. Furthemore, the observed running-related change of adiponectin in CSF, greater than expected based on the indicator of BBB/BCB permeability, also points at a potential running-induced increase in adiponectin uptake by the brain. This could support the notion that adiponectin promotes exercise-induced brain plasticity, explaining to an extent neuroprotective effects of exercise in humans, by means of the active dialogue between body and the brain.

We acknowledge the limitations of our pilot feasibility study, which include a small number of participants as well as their gender and training heterogeneity, and we plan to increase the number of volunteers. Changes in CSF/serum cytokine levels could occur in other than selected time points limiting thus our ability to detect running-induced changes.

## Conclusion

An acute bout of high-intensity aerobic exercise (90-min running) distinctly modified levels of adiponectin and to a smaller extent also other 16 cytokines as well as insulin and albumin in cerebrospinal fluid of healthy young volunteers, in association with cognition, physical fitness and metabolism. This indicates a possibility that specific exercise-regulated bioactive molecules in cerebrospinal fluid could be implicated in an adaptive crosstalk between peripheral tissues and the brain, mediating the beneficial effects of exercise on the brain by regulating appetite/energy metabolism, neuroprotection and cognition.

## Methods

### Ethical approval

The study protocol was approved by the Ethics Committee of the University Hospital Bratislava and conforms to the ethical guidelines of the *Declaration of Helsinki 2000*. All the participants received detailed information on the study protocol and signed written informed consent prior entering the study.

### Study population

Eleven (6/5 M/F) young (24.6 ± 5.1 [18–34] years of age) regularly physically active (≥1-h of intense, mostly aerobic physical exercise: running, cycling, swimming, ≥3x/week) nonsmokers, without a history of chronic diseases or regular pharmacotherapy were recruited.

### Study design

The study protocol consisted of two experimental days: Day 1 (90-min run), completed by all eight volunteers and Day 2 (baseline), completed by six volunteers; (depicted in greater detail in Supplementary Fig. 1).

In the morning of Day 1, prior to the 90-min run, participants were subjected to cognitive testing (CogState&Memtrax). Next, a cannula was inserted into the cubital vein to collect blood at baseline (30-min post-insertion), as well as immediately after and 60-min after the run. Participants were fed one banana (~100 g) after the collection of the fasting blood sample and prior to the run. Participants ran on a treadmill in a gym (n = 2) or outside (n = 6, average outside temperature 10 ± 4.5 °C). Exercise intensity was monitored with Polar RS300X (Finland) and maintained at 75–80% of HRmax (average pace 6 min 8 s/km). Free access to water was provided during the entire run. A sample of cerebrospinal fluid (4–5 ml) was taken by atraumatic (pencil point) lumbar puncture 60-min after completion of the run. After lumbar puncture and the last blood draw (60-min after the run), study participants were allowed to take refreshments and were subjected to the cognitive testing (CogState&Memtrax) (Supplementary Fig. 1).

On the day 2 (baseline), blood was taken from a cubital vein 30-min after a cannula insertion. After recording anthropometric (quadrupedal bioimpedance), metabolic (indirect calorimetry) parameters and blood pressure, participants were subjected to a battery of standardized questionnaires. Cerebrospinal fluid (~4–5 ml) was collected by lumbar puncture in the hospital setting by an experienced neurologist.

The two experimental days were separated by ~4 weeks. Participants were asked to refrain from an exhaustive physical activity and alcohol for 48-h prior to the experiment.

Cardiorespiratory fitness was assessed by cycle spiroergometry on a separate day.

### Clinical and metabolic phenotyping

Body Mass Index (BMI) was calculated (body weight [kg]/height [m]^2^) and waist circumference measured at the mid-point between the lower border of the rib cage and the iliac crest. Body composition was assessed by quadrupedal bioimpedance [BF511, Omron, Japan]. Resting energy expenditure (REE) and metabolic substrate preference (RQ; respiratory quotient) were measured after an overnight fast and following 30-min bed rest with indirect calorimetry at supine position and at thermal neutrality [Ergostik, Geratherm Respiratory, Germany].

Blood pressure and heart rate were measured [Dimarson DM-14508, Taiwan].

Physical activity^80^, demographic and modified food preference questionnaire^81^ were used. The USDA food composition database was used for the assessment of a micronutrient profile for each food included in the food preference questionnaire. The individual preference for food containing specific micronutrient was calculated only if this micronutrient was present in sufficient quantity in more than 10 out of 70 food items.

### Cardiorespiratory Fitness

Cardiorespiratory fitness was assessed by cycle spiroergometry. Participants were instructed to refrain from exhaustive physical activity 48-h prior to the test. Maximal oxygen consumption (${\dot{{\rm{V}}}O}_{2}$ max) was determined from the continuous measurement of gas exchange [PowerCube - Ergo, Ganshorn Medizin Electronic GmbH, Germany] during an incremental exercise test [Lode-Corival cycle ergometer, Lode B.V., Netherlands] coupled with continuous ECG, blood pressure and heart rate monitoring [Schiller, AT-104 PC, Switzerland]. After 2-min resting period, volunteer was asked to pedal against the initial load of 50 Watts, with frequency of 60 strokes/min. Load was increased continually by 25 Watts/min to detect ${\dot{{\rm{V}}}O}_{2}$ max. At 250 Watts, the subject was allowed to increase the cycling frequency to 70 strokes/min. ${\dot{{\rm{V}}}O}_{2}$ max was attained when the participant was uncapable to continue cycling against continuously increasing load. Blood pressure, heart rate and gas exchange continued to be recorded during the next 6-min recovery period, while the volunteer was still seated on the cycle ergometer.

### Cognitive testing

Cognitive testing included a battery of computerized cognitive tests: (i) CogState (reaction time, attention, working memory) and (ii) Memtrax (reaction time, short term visual memory). Participants were familiarized with the testing battery 7–14 days prior to the experiment. Cognitive tests were administered by an experienced investigator in a calm environment with an appropriate lighting.

The CogState testing battery consisted of 4 playing card memory tasks (Detection Task, Identification Task, One Card Learning Task, One Card Back Task). For each task, a participant single-handedly operated mouse to answer YES or NO by clicking the right and the left mouse button, respectively. Raw score for each task was transformed automatically by the CogState software to generate a composite score considering both answer accuracy and reaction time^82^. Unlike in classical cognitive pentests, the computerized CogState battery testing is designed for repetitive use without risking the possibility that participat remembers the task.

MemTrax is a visual short-term memory test composed of series of catching pictures. Participants were instructed to press the space button on the keyboard in case they remember seeing the picture on the screen before. Software records both response accuracy and reaction time^83^.

### Sampling of cerebrospinal fluid

Lumbar puncture (LP) was performed in a bended left lateral decubitus position using [Sprotte®Pencil-Point needle, 22 G]. This technique was used to reduce postpuncture headache also by others^84,85^. We have provided sufficient post-puncture hydration and a small high-fat/high-glucose snack after sampling. Post-puncture headache was reported after two out of twenty individual punctures performed in the frame of this study, with a spontaneous resolution within 1–5 days. Samples of CSF were collected to polypropylene tubes, immediately placed on ice and centrifuged (400 × g, 10-min, 4 °C) to dispose of cells. Aliquots of CSF were stored at −80 °C. Volunteers were asked to minimize their physical activity for at least 24-h after the procedure. The CSF was always sampled at the same time of the day using the same sampling procedure and sample processing techniques. Previous studies showed a good level of stability for proteins in cerebrospinal fluid^86^. For ethical reasons we have not performed repeated baseline CSF sampling (Supplementary Fig. 1). Importantly, there was virtually no difference in baseline levels of serum adiponectin, lactate, creatine kinase, albumin, glucose, insulin and HOMA-IR (Supplementary Table 2) between the two experimental days, despite the necessary 4-week distance. In addition, the levels of cytokines and metabolites in CSF at baseline fasting conditions are known to be maintained at a relatively narrow physiological range and the blood CSF barrier function is also well maintained in healthy young individuals^87^. The fact that only about 10% of proteins in CSF (17 out of 174 of proteins detected by antibody array) were significantly changed with the acute running exercise (Supplementary Fig. 1) advocates for a distinct exercise-related regulation.

### Biochemical analyses

Serum and CSF levels of glucose [glucose-hexokinase kit], albumin [N antiserum company], insulin [ADVIA Centaur Insulin, all from Siemens Healthcare Diagnostics, UK] were analyzed in a certified laboratory (Alfa-Medical, Slovakia). HOMA-IR was calculated (fasting insulin (μU/l) × fasting glucose (nmol/l)/22.5.

### Antibody array

A sample of CSF (500 μl) was mixed 1:1 with Cell Lysis Buffer [RayBio] containing protease inhibitors (Complete Mini [Roche, Germany], leupeptin, pepstatinA and aprotinin [SigmaAldrich, USA]) and phosphatase inhibitor coctail [SigmaAldrich, USA]. Lysis continued during 30 min inversion mixing at RT in polypropylene protein LB tubes [Sarstedt, German]. Next, Odyssey blocking buffer was added to a final volume of 1.2 ml. After 1-h blocking [Odyssey Blocking Buffer, Li-COR, USA with 0.05% Tween20], antibody pre-printed membranes [Human Cytokine Array C2000, RayBio, USA] were incubated with the sample overnight at 4 °C. Membranes were washed, incubated with a mix of biotin conjugated anti-cytokine antibodies 1-h/RT, washed and incubated with IRDye streptavidin solution for 45-min/RT protected from light. All incubations and washings were done on a shaking platform to ensure equal liquid dispersion. Membrane images were obtained by Odyssey Infrared Imaging System [Li-COR, USA] and protein signal intensity was measured. The average intensity of signal for each cytokine was normalised to an internal positive control on each membrane.

### ELISA

Adiponectin levels in serum and CSF were determined by high sensitivity Human ELISA [Biovendor, Czech Republic].

### Immunoblotting

CSF samples (20 μl) were mixed with loading dye, incubated at 96 °C for 5 min, separated on the SDS-PAGE (10%) and transferred to a PVDF membrane [Millipore, USA]. After 1 h blocking with Odyssey Blocking Buffer [LI-COR, USA], adiponectin was detected with mouse monoclonal anti-Adiponectin (human) mAb (HADI 773) antibody [Adipogen, USA; dilution 1:1000], and visualized with highly cross-adsorbed goat anti-mouse IRDye 680RD antibody [LI-COR, USA, dilution 1:10000] using the Odyssey IR Imaging System [LI-COR, USA]. In parallel with CSF, samples of human adipose tissue (20 μg), muscle (40 μg) and serum (4 μl) were used to detect oligo- and multimeric forms of adiponectin (Fig. 1E). PageRuler Prestained Protein Ladder 10–180 kDa [Termo-Scientific, Lithuania] was used to determine apparent molecular weight of various forms of adiponectin.

### Statistical analyses

The effect of an acute exercise bout on the cytokine levels in serum and CSF was assessed using either Student’s paired t-test (two-time points) or multiple comparision testing with ANOVA and Tukey post-hoc test (three time-points) using JMP [version 4.0.4 academic; SAS Institute, USA]. Pearson linear correlation analysis model was used, in parallel to the stepwise multiple regression analysis model. Values are presented as mean ± SEM. The statistical significance was set at p < 0.05. Changes induced by an acute exercise were calculated as a fold change (levels after run/baseline levels).

## Supplementary information

Supplementary material
