# Supplementary material for: Effects of running on adiponectin, insulin and cytokines in cerebrospinal fluid in healthy young individuals
Source: Sci Rep. 2019 Feb 13;9:1959. doi: 10.1038/s41598-018-38201-2 (PMC6374465; doi:10.1038/s41598-018-38201-2)
Supplement: Supplementary file 1 — Supplementary material [file 41598_2018_38201_MOESM1_ESM.pdf]

Effects of running on adiponectin, insulin and cytokines in cerebrospinal fluid in healthy young individuals

Abbreviated title: Acute exercise modulates adiponectin in cerebrospinal fluid

Schön M.[1,2#], Janáková Z.[1,2#], Košutzká Z.[3], Nemec M.[1], Tomková M.[1], Jacková L.[1], Máderová D.[1], Slobodová L. [1,2], Valkovič P.[3], Ukropec J.[1], Ukropcová B.[1,2,4]*

[1]Institute of Experimental Endocrinology, Biomedical Research Center, Slovak Academy of Sciences, Dúbravská cesta 9, 94505 Bratislava, Slovakia; [2]Institute of Pathological Physiology, Faculty of Medicine, Comenius University, 81108; [3]2nd Department of Neurology, Faculty of Medicine, Comenius University, University Hospital Bratislava, 83305; [4]Faculty of Physical Education and Sports, Comenius University, 81469 Bratislava, Slovakia

*Corresponding author:
A/Prof. Barbara Ukropcová MD, PhD
Institute of Experimental Endocrinology
Biomedical Research Center
Slovak Academy of Sciences
Dúbravská cesta 9, 94505 Bratislava
barbara.ukropcova@savba.sk

[#]Authors equally contributed to this work

Supplementary Table 1

adipocytokine	p value	% change	adipocytokine	p value	% change	adipocytokine	p value	% change	adipocytokine	p value	% change
IGF-II	0.004	-5.49	BMP-5	0.175	-5.35	MMP-9	0.336	-5.56	IL-3	0.633	1.32
Adiponectin	0.009	-21.31	NAP-2	0.175	-5.30	Amphiregulin	0.340	-9.51	bFGF	0.636	-2.24
Thrombopoietin	0.013	-2.05	PDGF R α	0.177	-10.09	FGF-9	0.341	-3.95	IL-1b	0.637	2.29
IL-13 R α 2	0.017	-4.84	Prolactin	0.178	-14.41	MIF	0.348	-1.46	Angiopoietin-2	0.673	-1.85
IL-18 R β	0.019	-10.58	IGF-I SR	0.179	-4.85	IL-13	0.349	5.08	IL-2 Ra	0.678	-0.54
TGF-b3	0.024	-3.65	NGF-R	0.179	-2.63	IL-12 p70	0.363	-4.16	CCL-28	0.684	-0.57
IL-18 BP α	0.026	-5.62	Erb83	0.185	-7.28	Fas Ligand	0.367	-1.78	Fit-3 ligand	0.687	-1.08
MIG	0.028	-8.42	IL-10 R β	0.188	-1.66	RANTES	0.374	-1.56	TECK	0.689	-1.13
PDGF AA	0.032	-10.11	GITR	0.189	-8.60	EGF	0.375	9.12	Leptin	0.690	-2.27
Activin A	0.033	-6.38	GRO	0.193	17.28	Cardiotrophin-1	0.379	-2.83	BMP-6	0.692	0.40
TGF β 2	0.037	-6.64	CD14	0.196	-5.46	IGFBP-6	0.397	-3.78	IL-10	0.694	2.86
MPIF-1	0.040	-2.84	Tie-2	0.196	-6.23	IL-2 R χ	0.401	-2.60	Dtk	0.709	0.33
IL-2	0.045	4.59	IGFBP-2	0.197	-4.50	SDF-1 β	0.401	-3.69	IL-1a	0.712	-1.79
IL-5 R α	0.047	-9.03	HCC-4	0.209	-3.87	TRAIL R4	0.405	-2.33	IL-21 R	0.718	0.59
LAP	0.048	-9.01	Angiogenin	0.211	-15.27	VEGF R2	0.409	-4.06	I-309	0.722	-1.10
Tie-1	0.049	-6.63	IGF-1	0.214	-1.35	ICAM-2	0.425	-2.06	MCP-1	0.729	-3.11
MMP-13	0.049	-7.13	SDF-1	0.216	-4.04	MIP-1a	0.425	-2.11	FGF-4	0.738	-0.62
GCP-2	0.051	-6.32	Leptin R	0.219	-4.43	GRO-a	0.434	0.34	SCF	0.743	-0.70
CXCL-16	0.055	-4.65	CK b8-1	0.234	4.79	ALCAM	0.435	-2.80	MSP-a	0.763	-0.62
Eotaxin-2	0.059	-4.38	Eotaxin	0.235	6.20	PIGF	0.448	-2.91	Fractalkine	0.768	-0.94
L-Selectin	0.072	-5.73	MDC	0.235	4.25	BDNF	0.457	3.01	GDNF	0.777	-0.71
VEGF R3	0.077	-5.56	PARC	0.236	-3.07	Eotaxin-3	0.459	2.13	TIMP-1	0.791	-1.07
IL-15	0.079	-4.48	M-CSF	0.237	3.21	FGF-6	0.462	5.34	FGF-7	0.799	0.04
Endoglin	0.092	-2.87	MMP-1	0.243	-2.94	PECAM-1	0.462	-4.51	IL-1 RI	0.799	0.82
LIF	0.093	-8.19	EGF-R	0.257	12.43	HGF	0.468	-4.24	IL-5	0.801	0.63
MCP-2	0.093	3.35	IL01ra	0.268	2.71	Fas/TNFRSF6	0.470	-2.25	IL-17	0.812	3.50
VE-Cadherin	0.098	-9.02	VEGF	0.272	-3.31	IL-11	0.478	-4.15	M-CSF R	0.813	0.15
IL-2 R β	0.101	-5.08	IL-12 p40	0.272	-3.00	BTC	0.484	-4.31	TARC	0.820	0.22
IP-10	0.103	-7.02	B7-1 (CD80)	0.276	-1.97	I-TAC	0.487	2.23	ENA-78	0.826	1.41
IL-9	0.108	-6.03	IGFBP-3	0.276	-6.06	VEGF-D	0.488	-1.85	MIP-1b	0.833	3.37
SCF R	0.108	-7.55	Lymphotactin	0.276	7.72	PDGF-BB	0.509	7.03	E-Selectin	0.838	-0.75
PDGF R β	0.141	-12.33	TRAIL R3	0.279	-4.50	TNF-b	0.511	-1.92	IL-6 R	0.839	-0.51
PDGF-AB	0.114	-10.88	MCP-3	0.282	3.70	LIGHT	0.517	-1.52	IL-7	0.871	-1.21
IGFBP-4	0.126	3.84	DR6 (TNFRSF21)	0.291	-2.80	TIMP-2	0.519	-3.26	GCSF	0.876	5.15
MCP-4	0.126	4.03	uPAR	0.291	7.24	sTNF RII	0.523	3.63	TGF-b1	0.891	2.49
IL-1 R4/ST2	0.130	8.05	MIP-3b	0.292	5.64	IL-4	0.528	4.00	GM-CSF	0.900	0.12
MIP-1d	0.134	-6.44	BMP-7	0.295	-3.80	IFN-G	0.530	-0.49	IL-6	0.907	0.15
NT-4	0.140	-4.88	IL-1 R II	0.295	-4.91	ICAM-3	0.535	0.65	IGFBP-1	0.919	2.11
IL-8	0.141	11.86	MMP-3	0.296	-3.23	NT-3	0.540	1.38	IL-16	0.989	1.64
Osteoprotegerin	0.145	-6.04	sTNF RI	0.310	10.60	Oncostatin M	0.543	-1.01	BMP-4	0.992	1.52
AgRP	0.146	-5.30	MIP-3A	0.311	-3.12	TNF-a	0.550	1.58	Siglec-5	0.995	1.92
Axl	0.151	9.56	sgp130	0.317	5.66	CTACK	0.551	-2.24	ICAM-1	0.999	1.21
CNTF	0.158	5.01	TGF α	0.327	-4.74	BLC	0.580	-0.32			
GITR-Ligand	0.172	-5.27	b-NGF	0.329	-6.11	TIMP-4	0.581	3.93			

Supplementary Table 1: *The list of all 174 adipocytokines assessed in paired CSF samples by protein arrays (n=6) arranged according to the level of significance (p-value, paired T-test).*

Supplementary Table 2

	Exp. day 1	Exp. day 2	p value
fasting adiponectin (mg/L)	3.35±0.28	3.47±0.19	0.56
fasting glucose (mmol/L)	4.76 ± 0.29	5.03 ± 0.52	0.37
fasting lactate (mmol/L)	1.24 ± 0.27	1.68 ± 0.93	0.31
fasting insulin (mIU/L)	5.32 ± 2.18	6.81 ± 3.27	0.47
fasting albumin (g/L)	44.34 ± 2.72	44.09 ± 4.29	0.86
fasting creatine kinase (umol/L)	2.91 ± 1.30	2.34 ± 0.99	0.33
HOMA-IR	1.14 ± 0.50	1.57 ± 0.91	0.42

Supplementary Table 2: *The comparison between the fasting levels of several biochemical parameters in serum taken at Day 1 and Day 2 (p-value, paired T-test).*

Supplementary Figure 1

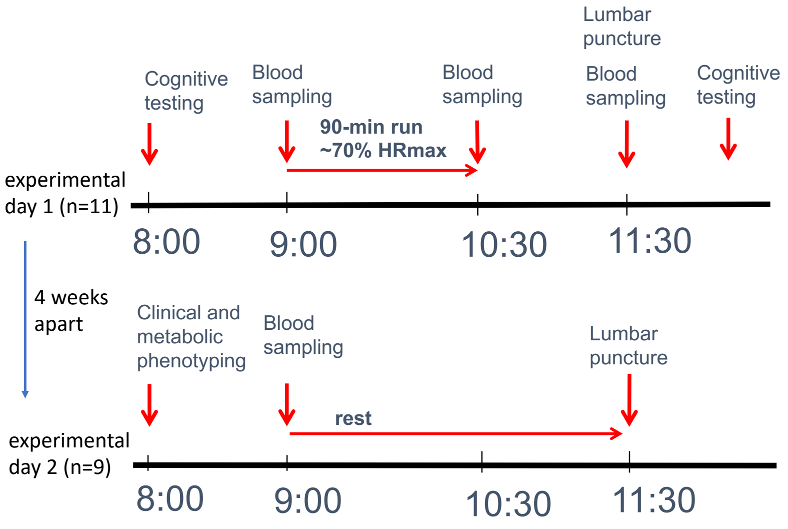

Supplementary Figure 1: *Study design. In the morning of experimental day 1. participants were subjected to the cognitive testing. Next, a cannula was inserted into the cubital vein to collect blood at baseline, as well as immediately after and 60min after the run. Participants were fed one banana (~100g) after the collection of the fasting blood sample and prior to the run. Exercise intensity was maintained at 75-80% of HRmax (maximal heart rate). A blood sample was collected immediately after the 90min run. A sample of cerebrospinal fluid was taken 60 minutes after completion of the run together with another blood sample. Study participants were then allowed to eat a carbohydrate snack, and next they were subjected to the post-exercise cognitive testing.*

On the experimental day 2. (baseline), blood was taken from a cubital vein 30min after a cannula insertion. After recording anthropometric, metabolic parameters and blood pressure, participants were subjected to a battery of standardized questionnaires. Cerebrospinal fluid was collected approximately at the same time as in the experimental day 1.

The two experimental days were separated by 4 weeks. Cardiorespiratory fitness was assessed by cycle spiroergometry on a separate day.
